# How does social enrichment produce health benefits?

**DOI:** 10.7554/eLife.43666

**Published:** 2018-12-03

**Authors:** Takefumi Kikusui

**Affiliations:** School of Veterinary MedicineAzabu UniversitySagamiharaJapan

**Keywords:** social enrichment, oxytocin, telomere length, novelty seeking, corridor field task, social neuroscience, Rat

## Abstract

Oxytocin appears to link social interaction and cell aging in rats, especially in females.

**Related research article** Faraji J, Karimi M, Soltanpour N, Moharrerie A, Rouhzadeh Z, Lotfi H, Hosseini SA, Jafari SY, Roudaki S, Moeeini R, Metz GAS. 2018. Oxytocin-mediated social enrichment promotes longer telomeres and novelty seeking. *eLife* **7**:e40262. doi: 10.7554/eLife.40262

In Japan, according to a recent survey by the Cabinet Office, 20% of elderly women and 25% of elderly men often feel lonely in everyday life because they have little social contact with other people. Indeed, 25% of the elderly have nobody who they can rely on when they have health problems, which has led to a substantial increase in the number of solitary deaths over the past 20 years. Many other countries report similar problems.

When social animals, including humans, live in isolation, their mental and physical health suffer (House et al., 1988). The results of a meta-analysis performed in 2010 indicated that people with stronger social relationships were 50% more likely to survive than those with weaker social relationships, regardless of age, initial health status or cause of death (Holt-Lunstad et al., 2010). This means that the influence of social relationships on the risk of death is comparable with some well-established risk factors, such as smoking and alcohol consumption, and exceeds the influence of other risk factors, including physical inactivity and obesity. However, the biological mechanisms that underlie the impact of social relationships are not yet well understood.

Rats are social animals, and previous studies have shown that their physical and mental abilities are better when they live in socially enriched housing (Leggio et al., 2005; van Praag et al., 2000). Now, in eLife, Reza Moeeini of the Avicenna Institute of Neuroscience, Gerlinde Metz of the University of Lethbridge and co-workers – including Jamshid Faraji, who is also at Golestan University of Medical Sciences – report a molecular mechanism that enables social interactions to have beneficial effects on the cells of rats (Faraji et al., 2018).

Most cells in the body can regenerate to some extent, but not infinitely. The ends of chromosomes consist of a characteristic repeating sequence of DNA and various proteins: the length of this telomore is one of the most reliable indicators of cell aging. With each cell division the telomeres shorten, and when the telomeres become shorter than a certain length, the cell stops dividing and enters a state called cell senescence (Shammas, 2011). Decreasing the cell's regenerative capacity reduces how well organs work, resulting in an increased risk of death.

To investigate the effects that living socially has on behavior and cell aging, Faraji et al. housed groups of 10 or 11 rats of the same sex together in a large area. After that, individual rats underwent a behavioral test in which they were placed into an unfamiliar field with a hiding space and an open area containing an object they hadn’t seen before. The researchers compared the behavior of these rats with the behavior of rats who had been housed in less social groups of just two or three animals.

Rats raised in the social group explored the new area more than those raised less socially. They also showed more novelty seeking behavior (measured through the length of time the rats spent in new zones), which is usually high in younger rats. Female rats showed a much greater increase in this behavior than male rats.

To detect the biological impacts of social enrichment, Faraji et al. examined skin cells and blood from the rats. An examination of the cells revealed that rats – especially females – who live in a complex social environment have longer telomeres. This is a breakthrough discovery that suggests that enriched social interactions can prolong the life of cells.

Faraji et al. also found that the blood of both male and female rats raised in socially enriched environments contained increased levels of oxytocin. Oxytocin is already known for its roles in regulating social bonds between mother-infant and male-female pairs (Donaldson and Young, 2008), and for its physical effects, such as reducing blood pressure and relaxing the movement of the intestinal tract (Uvnäs-Moberg, 1998).

To test whether oxytocin is linked to the protection of telomeres, Faraji et al. treated social rats with a compound that prevents oxytocin from acting on the body. In female rats, this resulted in cells having much shorter telomeres, and also reduced the extent of their novelty seeking behavior. Therefore, Faraji et al. conclude that social interaction stimulates the oxytocin system and through this mechanism preserves telomere length. These effects appear to be gender specific: blocking oxytocin did not have a significant effect in male rats.

These results make an important contribution to ‘social neuroscience’, an area of research that bridges biology and psychology. The late John Cacioppo, who founded this area with his colleague Gary Berntson, once explained that his interest in the biological and behavioral impact of social relationships was based on his experience of surviving a near-fatal car crash: this taught him that love and social connections are what really matter in life. By clarifying part of the mechanism behind how social interaction impacts the brain and body, Faraji et al. show just how important these connections really are.

It is also noteworthy that this study is the result of a collaboration between researchers from two countries, Iran and Canada. In the words of Peggy Mason of the University of Chicago, who was the Reviewing Editor for the paper: “This collaboration is an instantiation of many of the social connectedness ideas suggested by the paper. And a somewhat remarkable one given our socially fraught world”.

**Figure 1:**
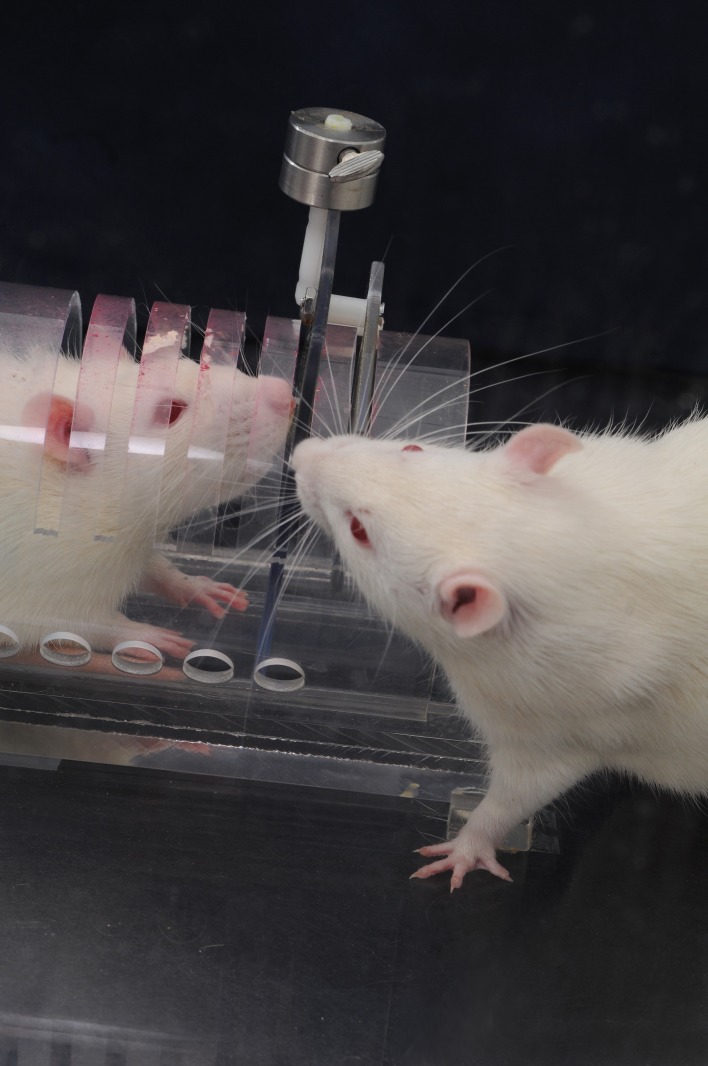
Rats raised socially seek out more new experiences and their cells age more slowly.

IMAGE CREDIT: Peggy Mason
